# Optimizing Total Joint Arthroplasty for Patients Living With Human Immunodeficiency Virus

**DOI:** 10.7759/cureus.28806

**Published:** 2022-09-05

**Authors:** Teja Yeramosu, Benjamin Chiang, Brandon Barnes, Jibanananda Satpathy

**Affiliations:** 1 Orthopaedic Surgery, Virginia Commonwealth University School of Medicine, Richmond, USA; 2 General Surgery, Riverside University Health System Medical Center, Moreno Valley, USA; 3 Orthopaedic Surgery, Baylor Scott & White Health, Dallas, USA

**Keywords:** postoperative complications, albumin, preoperative optimization, total joint arthroplasty, hiv

## Abstract

Introduction

Significant advancements in human immunodeficiency virus (HIV) treatment have led to an increasing life expectancy among patients living with HIV (PLWH). Given this rise in life expectancy, as well as the ability to lead a more active lifestyle, the rate of total joint arthroplasty (TJA) in this population is increasing. Unfortunately, the current medical literature surrounding the safety and efficacy of TJA in this patient population is indeterminant. Therefore, the purpose of this study was to determine if optimization of PLWH prior to TJA would result in any changes in the incidence of postoperative complications and hospital length of stay (LOS) when compared to historically reported data.

Materials and methods

A retrospective study was performed of all PLWH 18 years and older who underwent either a primary total knee arthroplasty (TKA) or total hip arthroplasty (THA) between 2009 and 2019 at our academic institution. Medical records were reviewed for each patient to assess demographics, comorbidities, preoperative laboratory studies, operative details, length of hospital stay, complications, and follow-up time. Patients were optimized using our institution's current optimization guidelines: body mass index (BMI) less than 40 kg/m^2^, hemoglobin >12 g/dL, no tobacco use within 30 days of surgery, albumin >3.5 g/dL. Independent-sample t-tests and Pearson's chi-square tests were used to evaluate the continuous and categorical variables, respectively.

Results

This study included 47 TJA in PLWH, including 14 TKA and 33 THA. Out of the 47 patients, 13 (27.7%) were fully optimized for all four variables: BMI, hemoglobin, non-smoking status, and albumin. There was no significant difference between the group of PLWH that was completely optimized and the group that was not in any patient characteristics, preoperative labs, intraoperative variables, or postoperative variables, including length of hospital stay and complications. A larger proportion of patients not completely optimized was found to be active smokers (p=0.0003). All complications occurred in cases in which the patients were not fully optimized. Subgroup analysis of PLWH, who were completely optimized, showed an average LOS of 4.3+/-1.5 days following TKA and 2.9+/-1.1 days following THA. Subgroup analysis of PLWH not completely optimized showed that each case was optimized for at least one variable and that those optimized for albumin had the largest (12.2%) number of complications.

Conclusion

PLWH can achieve a low rate of complications and LOS similar to that of the general population if medically and nutritionally optimized. Additional research is necessary to reveal well-defined parameters for achieving a higher rate of optimization prior to surgery in this important patient population.

## Introduction

Despite concentrated efforts to address this severe public health issue, the human immunodeficiency virus (HIV) continues to be a major global health tragedy. While many countries have started to witness a decrease in the incidence of HIV, the United States has been experiencing a rise in new cases since 2010, with a current prevalence of approximately 0.2% of the entire United States population [1]. Major advancements in the treatment of HIV, such as highly active antiretroviral therapy (HAART), have led to an increase in life expectancy amongst this patient population, as well as the ability to maintain an active lifestyle. Furthermore, patients living with HIV (PLWH) are thought to be at a higher risk of developing osteonecrosis of both the hip and the knee [2,3]. As osteonecrosis can cause the bones surrounding the hip and knee to collapse, resulting in significant pain and decreased function, total joint arthroplasty (TJA) is generally the best treatment [4]. Given the rise in life expectancy as well as the increased risk for potential osteonecrosis, the demand for TJA in PLWH will likely increase.

Advancements in HIV medication and surgical devices and techniques have allowed TJA to become safer for PLWH; however, the literature surrounding the efficacy of TJA in this patient population is uncertain. Several small retrospective studies show high rates of postoperative complications in PLWH following TJA, especially infection, as well as a longer length of hospital stay (LOS) [5-9]. However, many studies report no increased risk of overall postoperative complications, concluding that HIV is not an independent risk factor for infection if PLWH is on HAART [10-14]. Therefore, the current rate of complications following TJA, and interventions and protocols that may further reduce postoperative complications in PLWH, are still unclear. 

In order to develop an evidence-based, standardized, preoperative optimization protocol to be used prior to total hip arthroplasty (THA) or total knee arthroplasty (TKA) with the objective of improving surgical outcomes for patients, Johns et al. performed a review of the literature [15]. They found that screening for various medical and nutritional preoperative risk factors was associated with a significant reduction in prosthetic joint infection (PJI) as well as a significantly shorter mean hospital LOS, mean cost of care, and hospital readmission rates. While this inexpensive intervention resulted in improved outcomes, this protocol has not been evaluated in the setting of TJA for PLWH.

The purpose of this study was to determine whether medical and nutritional optimization of PLWH prior to TJA will result in a rate of postoperative complications and LOS similar to that of the general population. We hypothesize that PLWH who are optimized accordingly prior to TJA will have comparable rates of complications and a similar LOS to the general population.

## Materials and methods

This study was approved by the university Institutional Review Board. A retrospective study was performed of all PLWH 18 years and older who underwent either a primary TKA or THA between 2009 and 2019 at our academic institution. All relevant cases were identified by using Current Procedural Terminology (CPT) and International Classification of Diseases (ICD-10) codes for primary TKA and THA, and HIV, respectively.

Medical records were reviewed for each patient to assess demographics, comorbidities, preoperative laboratory studies, operative details, length of hospital stay, complications, and follow-up time. Demographics and comorbidities include age, sex, body mass index (BMI), coronary artery disease (CAD), congestive heart failure (CHF), pulmonary disease (defined as either obstructive sleep apnea or chronic obstructive pulmonary disease), diabetes, smoking status, hypertension, inflammatory state, anemia, drug use, and hepatitis. Preoperative laboratory studies include levels of hemoglobin A1c, albumin, transferrin, hemoglobin, creatinine, CD4 T cell count, and HIV viral load. Operative details include estimated blood loss and operative time. Lastly, complications include infection, fracture, neurologic injury, wound healing, acute kidney injury (AKI), and dislocation. Patients were excluded from our study if they were prisoners, had coagulopathy, or if they had a postoperative follow-up of fewer than 90 days.

Patients were optimized using our institution's current optimization guidelines: BMI less than 40 kg/m^2^, hemoglobin >12 g/dL, non-smoking status or no tobacco use within 30 days of surgery, albumin >3.5 g/dL. Albumin was used as a surrogate for nutritional status, while the other variables were used to approximate medical status. These parameters are very similar to those described by Johns et al., with the only difference being that they also recommend a measure of glucose control (hemoglobin A1c <7.0-7.5%) and negative methicillin-resistant Staphylococcus aureus colonization status [15]. Patients who met all the criteria were classified as fully optimized, and patients who met at least one of the criteria for optimization were classified as partially optimized. Patients who did not meet any of the criteria were classified as not optimized.

Independent-sample t-tests and Pearson's chi-square tests were used to evaluate the continuous and categorical variables, respectively. An alpha of 0.05 was used for statistical significance. All analyses were completed using JMP Software (Version 15.0, SAS Institute Inc., Cary, NC).

## Results

This study included 47 total TJA in PLWH, including 14 TKA and 33 THA. All patients were on HAART therapy at all time points. The patients had an overall mean age of 53.06 years with a mean follow-up of 32 months. Patients tended to be overweight, with a mean BMI of 28.5 +/- 6.5 kg/m^2^, and had a range of comorbidities, the most common of which were hypertension (46.8%), diabetes (21.3%), and hepatitis (17.0%). There were a total of seven complications (14.9%) in four patients. One patient who underwent a complex TKA (valgus and flexion contracture) developed a peroneal nerve palsy. Postoperatively, the patient fell and sustained partial wound dehiscence and patella fracture that required revision surgery which was subsequently complicated by infection. The overall mean LOS was 87.90+/-40.16 hours or 3.7+/-1.7 days.

Thirteen out of the 47 cases (27.7%) were fully optimized for all four variables: BMI, hemoglobin, smoking status, and albumin. Between the group of PLWH that was completely optimized and the group that was not, there was no significant difference in any of the patient characteristics, preoperative labs, intraoperative variables, or postoperative variables, including length of hospital stay and complications (Table 1). A larger proportion of patients not completely optimized were found to be active smokers (p=0.0003). No other comorbidity was discovered to be significant between the two cohorts. All complications occurred in cases in which the patients were not fully optimized. Two patients, both of which were active smokers, required revision surgery, one for recurrent dislocation and another for periprosthetic joint infection.

**Table 1:** Patient characteristics, comorbidities, preoperative, intraoperative, and postoperative variables in PLWH who were completely optimized vs. PLWH who were partially optimized *Certain postoperative variables, including hematoma, DVT/PE, UTI, pneumonia, stroke, myocardial infarction, 30-day readmission, and 30-day mortality, were not seen in either group. BMI - body mass index; DVT/PE - deep vein thrombosis/pulmonary embolism; HAART - highly active antiretroviral therapy; PLWH - persons living with human immunodeficiency virus; UTI - urinary tract infection

Patient characteristics*	n	Completely optimized PLWH (n=13)	Partially optimized PLWH (n=34)	p-value
Age (years), mean (SD)	47	53.23 (7.33)	53.00 (8.45)	0.929
Male, n (%)	47	5 (38.46)	16 (47.06)	0.596
BMI, mean (SD)	47	29.43 (7.01)	28.11 (6.32)	0.529
HAART, n (%)	47	13 (100.00)	34 (100.00)	-
Comorbidities				
Diabetes, n (%)	47	1 (7.70)	9 (26.47)	0.159
Smoker, n (%)	47	0 (0.00)	20 (58.82)	0.0003
Hypertension, n (%)	47	8 (61.5)	14 (41.18)	0.211
Drug use, n (%)	47	0 (0.00)	1 (2.94)	0.532
Coronary artery disease, n (%)	47	0 (0.00)	1 (2.94)	0.532
Congestive heart failure, n (%)	47	1 (7.70)	0 (0.00)	0.102
Pulmonary disease, n (%)	47	1 (7.70)	5 (14.71)	0.519
Inflammatory state, n (%)	47	1 (7.70)	3 (8.82)	0.901
Anemia, n (%)	47	1 (7.70)	3 (11.77)	0.685
Hepatitis, n (%)	47	1 (7.70)	7 (20.59)	0.293
Preoperative labs				
Hemoglobin A1c (%), mean (SD)	15	5.30 (0.58)	5.70 (0.83)	0.37
Albumin (g/dL), mean (SD)	44	4.27 (0.29)	4.21 (0.46)	0.659
Transferrin (mg/dL), mean (SD)	11	262.75 (15.35)	281.00 (40.03)	0.399
Creatinine (mg/dL), mean (SD)	47	0.90 (0.24)	0.99 (0.21)	0.156
CD4 count, mean (SD)	39	669.63 (476.72)	590.86 (264.59)	0.503
Viral load (IU/mL), mean (SD)	39	120.78 (368.20)	2709.53 (10942.61)	0.641
Detectable viral load, n (%)	39	4 (36.4%)	8 (28.6%)	0.635
Intraoperative variables				
Estimated blood loss (mL), mean (SD)	47	261.54 (155.66)	307.35 (235.21)	0.513
Operative time (minutes), mean (SD)	26	95.60 (28.93)	114.33 (38.72)	0.316
Postoperative variables				
Length of stay (hours), mean (SD)	40	85.92 (34.02)	88.65 (42.79)	0.846
Infection, n (%)	47	0 (0.00)	2 (5.88)	0.372
Fracture, n (%)	47	0 (0.00)	1 (2.94)	0.532
Neurologic injury, n (%)	47	0 (0.00)	1 (2.94)	0.532
Wound healing, n (%)	47	0 (0.00)	1 (2.94)	0.532
Acute kidney injury, n (%)	47	0 (0.00)	1 (2.94)	0.532
Dislocation, n (%)	47	0 (0.00)	1 (2.94)	0.532
Revisions, n (%)	47	0 (0.00)	2 (5.88)	0.372
≥1 complication	47	0 (0.00)	5 (14.71)	0.1436

Subgroup analysis of PLWH, who were completely optimized, showed no complications following either TKA or THA. Furthermore, this group had an overall average LOS of 85.92+/-34.02 hours or approximately 3.6+/-1.4 days. Upon further splitting by surgery type, PLWH had an average LOS of 104.37+/-35.48 hours, or 4.3+/-1.5 days, following TKA and 70.55+/-26.16 hours, or 2.9+/-1.1 days, following THA.

**Figure 1:**
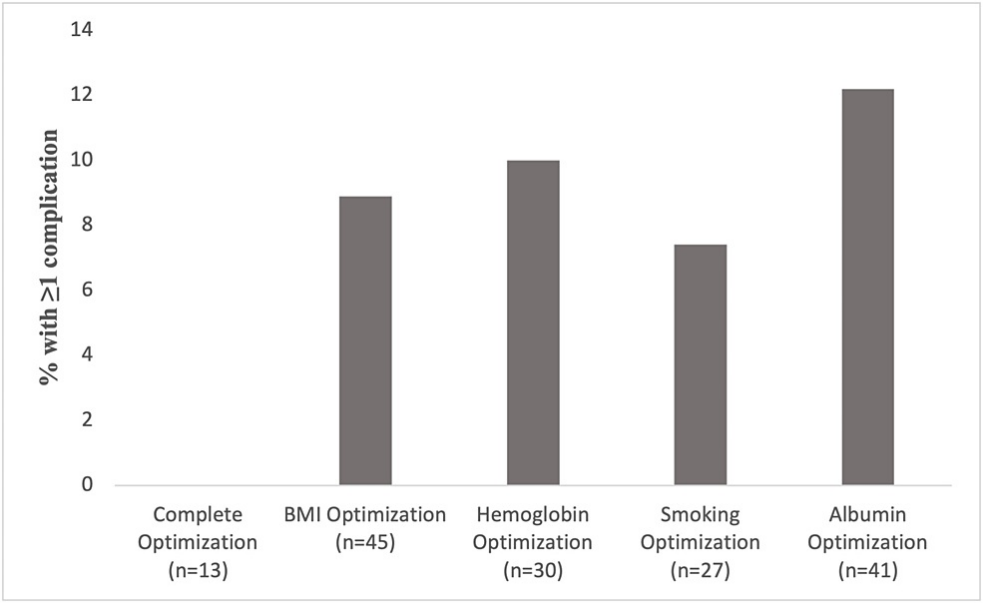
Percent of optimized patients with at least one complication BMI - body mass index

Subgroup analysis of PLWH not completely optimized showed that each case was optimized for at least one variable. Furthermore, the proportion of patients in this subgroup that developed at least one complication varied (Figure 1). Most of the cases (95.7%) were optimized for BMI, with 8.89% of these cases developing at least one complication; 63.8% of cases were optimized for hemoglobin, with 10% of these cases developing at least one complication; 57.4% of cases were optimized for smoking status, with 7.41% of these cases developing at least one complication; 87.2% of cases were optimized for albumin, with 12.2% of these cases developing at least one complication.

## Discussion

Advancement in antiretroviral therapy, along with other therapeutic interventions, has allowed PLWH to live longer, more active lifestyles, with many living past the age of sixty [16]. As this cohort of patients becomes older, the incidence of elective TJA will likely increase. Currently, however, there are limited and inconsistent data regarding outcomes following TJA in PLWH. While various evidence-based preoperative protocols have been shown to provide overall improved outcomes following TJA, few articles discuss methods of mitigating postoperative complications in the patient population of PLWH following TJA [15]. Therefore, the purpose of this study was to assess the outcomes of PLWH, who were optimized with a well-researched, evidence-based, preoperative protocol prior to TJA.

In our study, we had 47 cases that were each optimized for at least one of the criteria described by Johns et al., specifically, BMI, hemoglobin, smoking status, and albumin, which served as a surrogate for nutritional optimization [15]. Overall, we found that the rate of postoperative complications was approximately 14.9%, with infection being the most predominant at 4.3%. Furthermore, we found that the overall mean LOS was approximately 3.7+/-1.7 days following TJA. These results are similar to some previously published reports. Boylan et al. described an increased risk of wound infection and LOS in a large nationwide database study, identifying an infection rate of 9% and LOS of 4.8 days [8]. Similarly, Capogna et al. and Lin et al. report rates of deep infection at 4.4% and 9.1%, respectively, following TJA, but do not mention LOS [4,6,9].

In the subgroup of patients who were completely optimized, there were no complications, including infection. The rate of complications following TKA or THA is generally low, with fewer than one percent of patients typically experiencing deep vein thrombosis or pulmonary embolism, urinary tract infection, myocardial infarction, renal failure, pneumonia, and mortality [17]. Furthermore, the rate of PJI is highest during the first two years following surgery and typically ranges between 0.5% and 2% for TKA and 0.5% and 1.0% for THA [18]. As the mean follow-up in our study was greater than two years, it is likely that we were able to identify all patients in our cohort who had developed a PJI. Turcotte et al. also found that after stratifying by BMI, patients with a similar BMI as our cohort had a mean LOS of 2.2+/-3.3 days for TKA and 2.1+/-3.8 days for THA [17]. This is slightly lower than what was found in our study: 4.3+/-1.5 days following TKA and 2.9+/-1.1 days following THA, possibly due to the small sample size resulting in increased variability in our results. Interestingly, Enayatollahi et al. found that rates of PJI in PLWH and PLWH and hemophilia following TJA were 2.28% and 10.98%, respectively, concluding that the treatment of patients with HAART and optimization of underlying comorbidities appears to lower the rate of PJI in this patient population [14]. This conclusion is further supported by Lespasio et al., who stated that patients who have well-controlled HIV who receive HAART and have undetectable viral loads with a CD4+ T cell level above 200/m^3^ are at a similar risk of PJI as the average population, especially after medical and nutritional optimization [18].

The complications in this study were solely found in the cohort of PLWH who were not completely medically optimized; however, there was no significant difference in complications and LOS between the two cohorts. Although this is likely due to the small sample sizes, it is also possible that the absence of a significant difference is due to the fact that these patients were still partially optimized. When looking at the subgroup of PLWH, who were partially optimized in our study, cases with nutritional/albumin optimization developed the largest rate of complications (12.2%). However, Nelson et al. and Bohl et al. have previously found that hypoalbuminemia independently predicts complications after TJA [19,20]. This suggests that nutritional optimization is not enough to protect against postoperative complications following TJA, especially in PLWH. Another explanation for the lack of difference is that all patients were on HAART, which led to an undetectable viral load in the majority of patients in both cohorts. While few studies discuss practices for managing PLWH receiving TJA, one single-surgeon study by Gottschalk et al. found overall significantly reduced infection rates following TJA after implementing a protocol [21]. One element of this protocol involved all PLWH to be on antiretroviral therapy and be followed by the infectious disease team prior to operation.

Our study had limitations, mainly the small sample size and incompleteness of the data. Due to this, we were unable to follow the complete recommendations for optimization put forth by Johns et al. [15]. It is retrospective in nature which may bias our analysis. This is a single-institution study with numerous surgeons who have varying treatment preferences, and as such, the results may not be generalizable to other centers. However, having multiple surgeons may be an accurate representation of the varying treatment provided in the community. The strengths of this study include the long follow-up time, as well as the fact that there are few reports discussing optimization and protocols for TJA in this important patient population.

## Conclusions

In conclusion, our study suggests that PLWH can achieve a low rate of complications and a LOS similar to that of the general population if medically and nutritionally optimized. Optimization for all four guidelines can be difficult in this patient population, and further research may reveal more precise parameters and elucidate the role of achieving a higher rate of optimization prior to surgery.
